# Disulfidptosis: a new target for metabolic cancer therapy

**DOI:** 10.1186/s13046-023-02675-4

**Published:** 2023-04-27

**Authors:** Peijie Zheng, Chuntao Zhou, Yuemin Ding, Shiwei Duan

**Affiliations:** 1 Department of Clinical Medicine, School of Medicine, Hangzhou City University, Hangzhou, 310015 China; 2 Institute of Translational Medicine, Hangzhou City University, Hangzhou, 310015 China; 3 Key Laboratory of Novel Targets and Drug Study for Neural Repair of Zhejiang Province, Hangzhou City University, Hangzhou, 310015 China

## Abstract

Altered metabolism is a hallmark of cancer and presents a vulnerability that can be exploited in cancer treatment. Regulated cell death (RCD) plays a crucial role in cancer metabolic therapy. A recent study has identified a new metabolic-related RCD known as disulfidptosis. Preclinical findings suggest that metabolic therapy using glucose transporter (GLUT) inhibitors can trigger disulfidptosis and inhibit cancer growth. In this review, we summarize the specific mechanisms underlying disulfidptosis and outline potential future research directions. We also discuss the challenges that may arise in the clinical translation of disulfidptosis research.

**Supplementary Information:**

The online version contains supplementary material available at 10.1186/s13046-023-02675-4.

Metabolic reprogramming is one of the important hallmarks of cancer, which usually results in a high dependence of cancer cells on specific nutrients or metabolic pathways. Targeting cancer metabolism to selectively kill cancer cells has been widely adopted in the era of precision oncology [1]. RCD is of particular value in the field of cancer metabolic therapy. For example, cuproptosis, a copper-dependent RCD caused by toxic stress of lipoylated tricarboxylic acid (TCA) cycle proteins in mitochondria [2].

Recently, Xiaoguang Liu et al. revealed a metabolic-related RCD, disulfidptosis [3]. They found that high expression of solute carrier family 7 member 11 (SLC7A11) in kidney cancer cells accelerates nicotinamide adenine dinucleotide phosphate (NADPH) depletion in the cytoplasm under glucose starvation. This leads to an accumulation of disulfides that cannot be reduced, inducing disulfide stress and eventually disulfidptosis. SLC7A11 is responsible for cysteine uptake and high expression in kidney cancer cells mediates high rates of cystine uptake. The accumulation of disulfides such as cystine induces disulfide stress, which is toxic to cells. Normally, NADPH provides reducing power to counteract disulfide stress and maintain cellular homeostasis. However, under glucose starvation, NADPH production from glucose through the pentose phosphate pathway (PPP) is limited. SLC7A11-mediated uptake of cystine further depletes intracellular NADPH, leading to its depletion and the accumulation of disulfide molecules. This triggers the formation of disulfide bonds between actin cytoskeleton proteins and the collapse of the actin filament (F-actin) network, eventually leading to disulfide ptosis (Fig. 1).

**Fig. 1:**
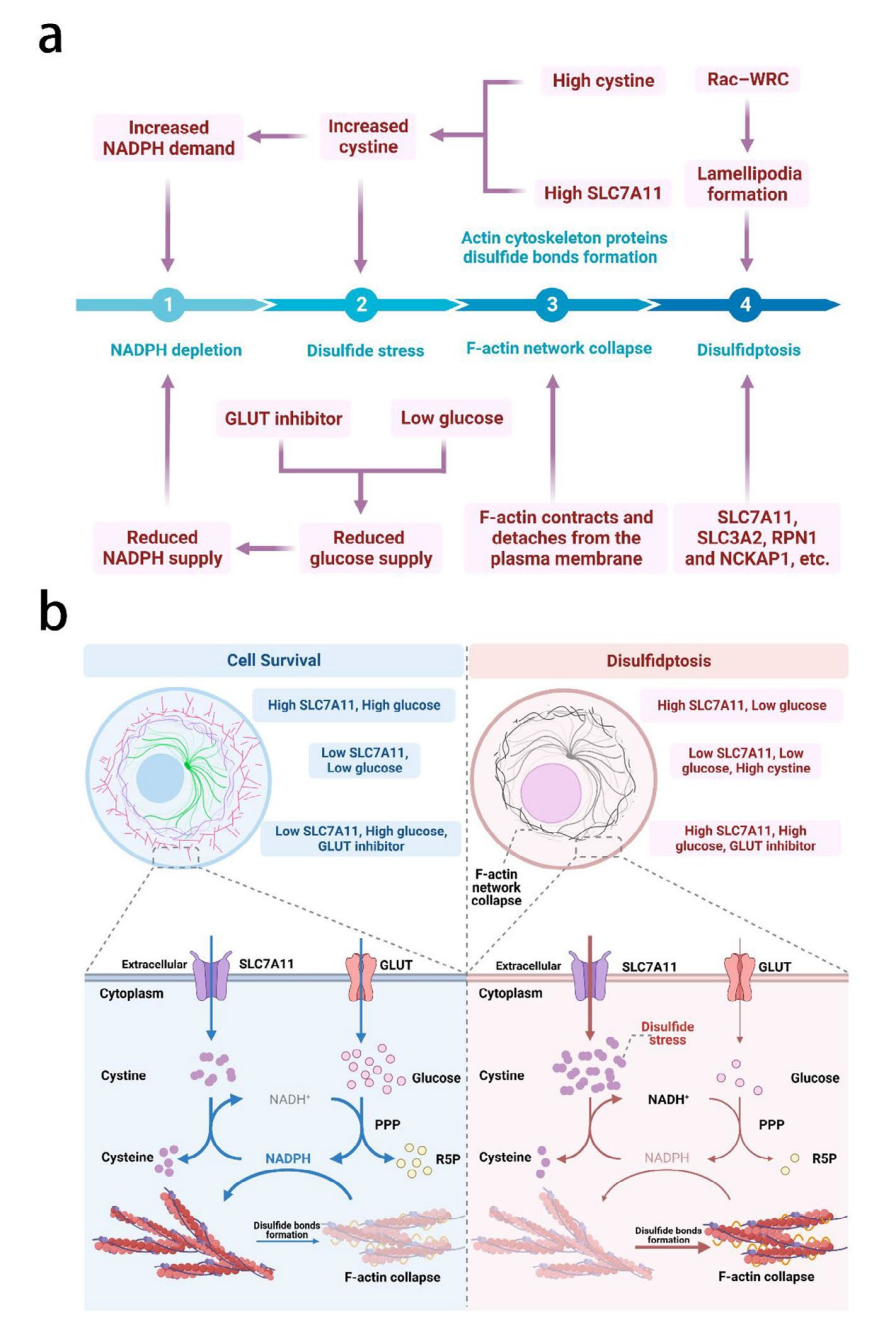
Schematic diagram of the mechanism of disulfidptosis **a**) The key to the occurrence of disulfidptosis is the disulfide stress caused by the accumulation of disulfides such as cystine. The reasons for the accumulation of disulfides include the high expression of SLC7A11 or the excessive intake of cystine caused by environmental homocysteine and the blockage of the reduction of cystine in the cell. Reduction of cystine requires NADPH. When intracellular NADPH is sufficient, excess cystine will be converted into cysteine, which will avoid the production of disulfide stress. The depletion of NADPH occurred before the production of disulfide stress, and NADPH is mainly the product of glucose PPP pathway metabolism. Starvation or the use of GLUT inhibitors will result in reduced glucose supply and thus NADPH supply. In addition, excess cystine also contributes to the depletion of NADPH. Afterward, under the pressure of disulfide stress, the formation of disulfide bonds between actin cytoskeleton proteins and the collapse of the F-actin network will occur, eventually triggering disulfidptosis. Among them, the collapse of the F-actin network involves F-actin contracts and detaches from the plasma membrane, while disulfidptosis is related to Rac–WRC-mediated lamellipodia formation. **b**) Intracellular NADPH is depleted by glucose starvation, GLUT inhibitors, or by providing excess cystine. This will trigger the production of disulfide stress in the cell, leading to the formation of disulfide bonds between actin cytoskeleton proteins and the collapse of the F-actin network, eventually triggering disulfidptosis. GADPH: Glyceraldehyde-3-phosphate dehydrogenase, GLUT: Glucose transporter, PPP: Pentose phosphate pathway, R5P: Ribose 5-phosphate, SLC7A11: Solute carrier family 7 member 11, WRC: WAVE regulatory complex

Xiaoguang Liu et al. examined whether kidney cancer cell death driven by SLC7A11^high^ under glucose starvation was a known type of cell death. They found that ferroptosis, apoptosis, necroptosis, autophagy inhibitors, and knocking out the essential genes of ferroptosis and apoptosis did not reverse this particular cell death (disulfidptosis). In addition, SLC7A11 overexpression also mildly restored the decreased ATP levels in kidney cancer cells under glucose starvation conditions, which ruled out the possibility that the cells died from ATP depletion. Through transmission electron microscope analysis, Xiaoguang Liu et al. also proved that cell death is not due to the toxicity of cystine crystal caused by the overexpression of SLC7A11. Interestingly, thiol-oxidizing agents (diamide and diethyl-maleate) significantly enhanced the death of SLC7A11^high^ cells under glucose starvation. In contrast, inhibition of disulfide accumulation prevented disulfidptosis, suggesting that intracellular accumulation of disulfide molecules is required for disulfidptosis.

Xiaoguang Liu et al. hypothesized that disulfidptosis could be triggered by the formation of disulfide bonds in protein molecules under NADPH depletion and disulfide stress. They quantified changes in disulfide bonds by using a bio-orthogonal chemical proteomic strategy under stable isotope labeling and found that glucose starvation can lead to at least a 1.5-fold increase in 90 cysteine sites. And Gene Ontology analysis revealed that related proteins that generate disulfide bonds are mainly enriched in biological processes related to actin cytoskeleton proteins. Furthermore, further non-reducing western blots determined that disulfide bond formation in actin cytoskeleton proteins is secondary to the depletion of NADPH and precedes disulfidptosis. Kidney cancer cells with high expression of the SLC7A11 gene were depleted of NADPH in 1 h under glucose starvation treatment, and the migration of actin cytoskeleton proteins was observed to be blocked in 2 h, which was related to the formation of disulfide bonds. The subsequent co-immunoprecipitation results strongly suggested the existence of intermolecular disulfide bonds. However, due to technical limitations, the authors were unable to identify interprotein disulfide-linked peptides. In addition, phalloidin staining also morphologically confirmed that the F-actin contract after glucose starvation treatment in kidney cancer cells with high expression of the SLC7A11 gene, thereby detaching from the plasma membrane of the cell, eventually leading to the occurrence of disulfidptosis.

To explore the regulatory relationship between genes, Xiaoguang Liu et al. conducted a genome-wide CRISPR-Cas9 screen and determined that Rac–WAVE regulatory complex (WRC)-mediated lamellipodia formation promotes disulfidptosis. The top four disulfidptosis-promoting genes were SLC7A11, solute carrier family 3 member 2 (SLC3A2), recombinant ribophorin 1 (RPN1), and NCK-associated protein 1 (NCKAP1), respectively. Among them, SLC3A2 encodes the SLC7A11 chaperone protein. The protein encoded by RPN1 is a subunit of N-oligosaccharide transferase located in the endoplasmic reticulum. Knockout of RPN1 also partially reverses disulfidptosis, but the specific mechanism is still unclear. NCKAP1 encodes Nck-associated protein 1, which is a component of WRC, but the reversal effect of NCKAP1 knockout on disulfidptosis is weaker than that of SLC7A11 knockout. This suggests that changes in the cytoskeleton may not be the only mechanism by which disulfidptosis occurs.

Finally, Xiaoguang Liu et al. also studied the anticancer effect of the targeted inhibitor of GLUT. The results demonstrated that both the GLUT1 inhibitor BAY-876 and the GLUT1 and − 3 inhibitor KL-11,743 were effective in inhibiting glucose uptake in UMRC6 kidney cancer cells. This process results in a reduction of NADPH production and an increase in the NADP+/NADPH ratio, which in turn leads to the abnormal formation of actin cytoskeletal protein disulfide bonds and the collapse of the F-actin network, and finally promotes the occurrence of disulfidptosis. In a similar manner, BAY-876 was effective in inhibiting the growth of SLC7A11^high^ UMRC6 kidney cell carcinoma xenografts in mice. It also showed efficacy in patient-derived xenograft (PDX) models that exhibited high expression of SLC7A11.

Research on disulfidptosis is still in its infancy, although it has yielded significant discoveries. To further understand the mechanism of disulfidptosis and its potential pathophysiological functions and promote its clinical translation, further research needs to be carried out in the following aspects in the future. (1) The specific mechanisms of disulfidptosis are not yet fully understood. It remains to be determined whether the collapse of the actin network is the only mechanism involved in disulfidptosis. (2) Current methods for inducing disulfidptosis are relatively limited. Future studies could explore whether disulfidptosis can be efficiently triggered by depleting intracellular NADPH, directly inhibiting the pentose phosphate pathway, or directly inducing disulfide stress. (3) Metabolic treatment of cancer cells may also affect non-cancerous cells, particularly immune cells. This may be one of the factors limiting the use of cancer metabolic therapy [1]. The induction of disulfidptosis through GLUT inhibition may not be immune to this effect, presenting a challenge for translational research on disulfidptosis. (4) The tumor suppressor effect of GLUT inhibitor monotherapy in preclinical models is weak and depends on the presence of high expression of SLC7A11 [4]. Biomarkers that detect SLC7A11 overexpression need to be optimized to identify patient populations suitable for GLUT inhibitor therapy. In addition, future studies are required to explore the anticancer effects of GLUT inhibitors in combination with other drugs.

Xiaoguang Liu et al. pioneered a form of metabolism-related cell death, which is expected to provide a new way for cancer metabolic therapy by inducing disulfidptosis to target the weakness of cancer metabolism. Furthermore, this study advances our understanding of the complex relationships between different RCDs. It has been suggested in the past that overexpression of SLC7A11 in cancer suppresses ferroptosis and thus plays a crucial role in promoting tumor growth [5]. The discovery of disulfidptosis subverts this traditional thinking, that is, SLC7A11 also plays an essential role in promoting disulfidptosis. Because SLC7A11 not only inhibits the occurrence of ferroptosis but also promotes disulfidptosis. Therefore, it is necessary to study whether the treatment that promotes ferroptosis by interfering with SLC7A11 will also inhibit the occurrence of disulfidptosis in the future. At the same time, it is necessary to study how to avoid this phenomenon in the targeted therapy of cancer. This will bring new thinking to future research related to cancer-targeted therapy.

## Electronic supplementary material

Below is the link to the electronic supplementary material.

Supplementary Material 1

## Data Availability

Data sharing is not applicable to this article as no new data were created or analyzed in this study.

## Abbreviations

F-actin: Actin filament
GADPH: Glyceraldehyde-3-phosphate dehydrogenase
GLUT: Glucose transporter
NADPH: Nicotinamide adenine dinucleotide phosphate
NCKAP1: NCK-associated protein 1
PDX: Patient-derived xenograft
PPP: Pentose phosphate pathway,
RCD: Regulated cell death
RPN1: Recombinant ribophorin 1
R5P: Ribose 5-phosphate
SLC3A2: Solute carrier family 3 member 2
SLC7A11: Solute carrier family 7 member 11
TCA: Tricarboxylic acid
WRC: WAVE regulatory complex
