# Supplementary material for: Disulfidptosis: a new target for metabolic cancer therapy
Source: J Exp Clin Cancer Res. 2023 Apr 27;42:103. doi: 10.1186/s13046-023-02675-4 (PMC10134647; doi:10.1186/s13046-023-02675-4)
Supplement: Supplementary file 1 — Supplementary Material 1 [file 13046_2023_2675_MOESM1_ESM.docx]

**Response to commentary for** **Disulfidptosis: a new target for metabolic cancer therapy (Manuscript Number: JECC-D-23-00400)**

Dear Editor and reviewers:

Thank you for your thoughtful and useful review of our manuscript and the opportunity to further improve our work by revision. We have made all the required changes according to the suggestion by the reviewers. Please find our point-by-point responses to all the inquiries below.

**Reviewer #1**

**Comment 1 : 1. The summary of Liu et al is succinct and clear, though some of the language could use editing for clarity. Specifically:
a. Lines 51-52 wording is incorrect; NADPH is reduced in all cells under glucose starvation, but SLC7A11 high cells are highly sensitive to this NADPH reduction.**

**b. Lines 53-54 wording is unclear; should be made clearer that SLC7A11 is the cysteine transporter responsible for uptake.**

**c. The wording should be made clearer on the reductive effects of NADPH, why that's needed with increased cysteine, and that it is produced from glucose.**

Response: Thank you for your valuable suggestions. We have revised the entire paragraph to provide a clearer description of the process leading to disulfidptosis. Please see the details below.

On page 3 , lines 18

High expression of SLC7A11 in kidney cancer cells accelerates NADPH depletion in the cytoplasm under glucose starvation. This leads to an accumulation of disulfides that cannot be reduced, inducing disulfide stress and eventually disulfidptosis. SLC7A11 is responsible for cysteine uptake and high expression in kidney cancer cells mediates high rates of cystine uptake. The accumulation of disulfides such as cystine induces disulfide stress, which is toxic to cells. Normally, NADPH provides reducing power to counteract disulfide stress and maintain cellular homeostasis. However, under glucose starvation, NADPH production from glucose through the pentose phosphate pathway is limited. SLC7A11-mediated uptake of cystine further depletes intracellular NADPH, leading to its depletion and the accumulation of disulfide molecules. This triggers the formation of disulfide bonds between actin cytoskeleton proteins and the collapse of the F-actin network, eventually leading to disulfide ptosis (Figure 1).

**d1. Other minor edits would include tidying up line 98-99 "****This reduces the supply of NADPH to promote the depletion of intracellular NADPH",**

Response: Thank you for the suggestion, after referring to the original we have rewritten this sentence Please check the details below:

On page 6 , lines 5

This process results in a reduction of NADPH production and an increase in the NADP+/NADPH ratio.

**d2.line 102 "influential in lung cancer PDX",**

Response: Thank you for the suggestion, we have amended this issue. Please check the details below:

On page 5 , lines 8

In a similar manner, BAY-876 was effective in inhibiting the growth of SLC7A11^high^ UMRC6 kidney cell carcinoma xenografts in mice. It also showed efficacy in patient-derived xenograft (PDX) models that exhibited high expression of SLC7A11.

**d3.and making the numbering schema in the paragraph starting at line 105 consistent (there's no (1)) and if necessary distinct from how references are presented.**

Response: Thank you very much for your suggestion. We have modified the numbering scheme in this paragraph.

**Comment 2 : The figure is visually attractive but contains an error in (B): the authors show the pentose phosphate pathway consuming NADPH to NADH+ and cysteine producing NADPH from NADP+; these are reversed. PPP produces NADPH and cysteine consumes it.**

Response: Thank you very much for your comment. We have fixed this error in Figure 1. Please check the details of Figure 1 below:

**Comment 3 : On line 114-115, I don't think its correct that GLUT is a target of disulfidptosis; it would be more correct to say that GLUT inhibition potentiates disulfidptosis.**

Response: Thank you for your suggestion. We have amended this issue. Please check the details below:

On page 6, lines 10

The induction of disulfidptosis through GLUT inhibition may not be immune to this effect, presenting a challenge for translational research on disulfidptosis.

**Reviewer #2**

**Comment 1 : I would just recommend the authors to do some language corrections before submitting for publication.**

Response: Thank you for your suggestions. We have carefully revised the language throughout the manuscript and hope that it now meets your standards for language proficiency.

**Comment 2 : Moreover it would be better to distinguish which GLUT transporter they are talking about, since Xiaoguang Liu et al do it in their work as well.**

Response: Thanks for your comment. We have addressed this issue by revising our references to the work of Xiaoguang Liu and colleagues. Please check the details below:

On page 5, lines 4

The results demonstrated that both the GLUT1 inhibitor BAY-876 and the GLUT1 and -3 inhibitor KL-11743 were effective in inhibiting glucose uptake in UMRC6 kidney cancer cells.

On page 6, lines 8

In a similar manner, BAY-876 was effective in inhibiting the growth of SLC7A11^high^ UMRC6 kidney cell carcinoma xenografts in mice. It also showed efficacy in patient-derived xenograft (PDX) models that exhibited high expression of SLC7A11.

Finally, we appreciate for Editor/Reviewers’ warm work earnestly and hope that the correction will meet with approval. Once again, thank you very much for your comments and suggestions.

Best regards,

Yours sincerely,

Shiwei Duan, Ph.D, Professor

School of Medicine,

Hangzhou City University,

Hangzhou, Zhejiang, China, 310015

Email: duansw@zucc.edu.cn

Phone & Fax: +86-571-88280195
